# Regulation of *ssb* Gene Expression in *Escherichia* *coli*

**DOI:** 10.3390/ijms231810917

**Published:** 2022-09-18

**Authors:** Isidoro Feliciello, Edyta Đermić, Helena Malović, Siniša Ivanković, Davor Zahradka, Sven Ljubić, Alfredo Procino, Damir Đermić

**Affiliations:** 1 Department of Clinical Medicine and Surgery, University of Naples Federico II, 81031 Naples, Italy; 2 Department of Plant Pathology, Division for Phytomedicine, Faculty of Agriculture, University of Zagreb, 10000 Zagreb, Croatia; 3 Division of Molecular Medicine, Ruđer Bošković Institute, 10000 Zagreb, Croatia; 4 Division of Molecular Biology, Ruđer Bošković Institute, 10000 Zagreb, Croatia

**Keywords:** SOS induction, basal *ssb* expression, UV and gamma irradiation

## Abstract

Bacterial SSB proteins, as well as their eukaryotic RPA analogues, are essential and ubiquitous. They avidly bind single-stranded DNA and regulate/coordinate its metabolism, hence enabling essential DNA processes such as replication, transcription, and repair. The prototypic *Escherichia coli* SSB protein is encoded by an *ssb* gene. Although the *ssb* gene promoters harbor an SOS box, multiple studies over several decades failed to elucidate whether *ssb* gene expression is inducible and SOS dependent. The SOS regulon is comprised of about 50 genes, whose transcription is coordinately induced under stress conditions. Using quantitative real-time PCR, we determined the *ssb* gene expression kinetics in UV- and γ-irradiated *E. coli* and revealed that *ssb* gene expression is elevated in irradiated cells in an SOS-dependent manner. Additionally, the expression of the *sulA* gene was determined to indicate the extent of SOS induction. In a mutant with a constitutively induced SOS regulon, the *ssb* gene was overexpressed in the absence of DNA damage. Furthermore, we measured *ssb* gene expression by droplet digital PCR during unaffected bacterial growth and revealed that *ssb* gene expression was equal in wild-type and SOS^−^ bacteria, whereas *sulA* expression was higher in the former. This study thus reveals a complex pattern of *ssb* gene expression, which under stress conditions depends on the SOS regulon, whereas during normal bacterial growth it is unlinked to SOS induction. The *E. coli ssb* gene is SOS regulated in such a way that its basal expression is relatively high and can be increased only through stronger SOS induction. The remarkable SOS induction observed in undisturbed wild-type cells may challenge our notion of the physiological role of the SOS response in bacteria.

## 1. Introduction

Genetic information is stored and protected within the double-stranded DNA molecule as a result of DNA duplex’s stability and inertness. However, the information encoded in the DNA duplex can only be used when DNA loses its duplex character, which occurs in a spatiotemporal manner during the obligatory physiological DNA metabolic processes of DNA replication, transcription, recombination, and repair. During these processes, enzymes, such as helicases and nucleases, act on the DNA duplex to transiently expose single-stranded DNA (ssDNA). The ssDNA intermediates are swiftly and tightly bound by the single-strand DNA binding (SSB) class of proteins in prokaryotes and RPA (replication protein A) proteins in eukaryotes [1,2,3,4,5,6], both of which play two essential roles in every cell: (a) SSB binds to ssDNA in a sequence-independent way and protects it from the activity of various nucleases [2,7,8,9,10]; (b) SSB interacts with/recruits a plethora of enzymes involved in DNA metabolism and thus regulates their activity, acting as a molecular matchmaker [11]. As a result of these activities, SSBs (RPAs) play a central role in DNA metabolism in organisms across all kingdoms of life and are, thus, essential proteins that enable fundamental cellular processes such as DNA replication, recombination, and repair [12].

Among the highly conserved bacterial SSB proteins, *Escherichia coli* SSB is considered prototypic regarding its structure and function [1,2,3,4,13]. The *E. coli* SSB protein exists as a homotetramer, with each monomer containing 178 amino acids, which can be divided into two domains based on proteolytic cleavage [13]. The well conserved N-terminal domain consists of 117 amino acids and is required for tetramer formation and cooperative ssDNA binding [13,14], while the C-terminal domain (especially its conserved C-terminal 9 residue acid tip) seems to mediate interactions with at least 14 DNA binding proteins that comprise the SSB interactome [11,15].

**Figure 1:**
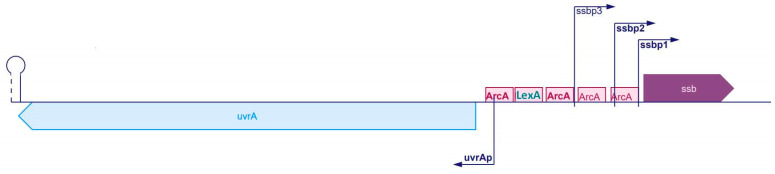
Location of LexA repressor binding sequence, (SOS) box, in relation to *ssb* and *uvrA* genes. ArcA is a sequence and onto ArcA a dual transcriptional regulator binds, which inhibits transcription during anaerobic conditions.

The essential SSB protein-coding gene, *ssb*, is located at centisome 92.08 of the *E. coli* chromosome [16]. The promoters of the *E. coli ssb* contain a single LexA repressor binding sequence (SOS box), which implies that *ssb* belongs to the SOS regulon [17]. The SOS regulon is a set of about 50 genes in the *E. coli* chromosome whose expression is repressed by a LexA repressor, until coordinately induced under stress conditions. The SOS response is induced when accumulated regions of ssDNA are bound by the RecA protein which acts as a coprotease to facilitate LexA repressor autocleavage [18]. Since SSB is such an important governor of DNA metabolism, it should not be surprising that it is a part of the regulon that regulates DNA replication, repair, mutagenesis, and cell division in bacteria under stress. However, the issue of SOS regulation of *E. coli ssb* expression turns out to be far from settled. Namely, *ssb* is regulated in a rather complex manner, containing three different promoters, of which LexA may repress only one, whereas the other two enable constitutive, though relatively low, *ssb* expression [17]. The LexA binding box is rather distant (−170 nt) from the *ssb* coding region and is divergent from the consensus SOS box (heterology index, HI, 6.98) [19]. Furthermore, the SOS box is situated in an intergenic region between the *ssb* and *uvrA* genes, which are transcribed in opposite directions, indicating that it can control the expression of both genes [19] (Figure 1).

Whether *E. coli ssb* is inducible under the SOS response was explored by several laboratories in the 1980s and then again in the 2000s. In these earlier studies, no increase in SSB level upon SOS induction were observed by radioimmunoassay [20,21,22,23]. On the other hand, two studies reported a very moderate and slow increase in SSB production in cells with a heavily induced SOS response [23,24], raising in turn the question of the physiological relevance of those results. When the *ssb* gene was cloned into lambda phage and introduced into SOS-noninducible cells, the uninterrupted synthesis of the SSB protein indicated that the *ssb* is not repressed by LexA, unlike *uvrA* [25].

The results of gene fusion investigations were also contradictory as one study failed to confirm SOS regulation of *ssb* [21], whereas the other showed that *ssb* expression is indeed SOS regulated [26]. However, Brandsma et al. cloned the gene fusion into a pBR322 plasmid so it was present in multiple copies in a cell, which further increases during SOS induction [27], hence making this experimental system not particularly useful since it does not mimic normal physiological conditions.

Another attempt at resolving the question of the *ssb* gene’s regulation was made possible by the introduction of DNA microarray assays [28]. Two studies used UV and one mitomycin C as an SOS inducer, and changes were measured in the genome-wide gene expression of the *E. coli* ORFs in response to DNA damage. Many discrepant results reported in the three studies notwithstanding, all three failed to detect an increase in *ssb* expression in treated cells [29,30,31]. This suggests that either the *E. coli ssb* is not regulated by the LexA repressor (and hence may not be a functional part of the SOS regulon), or the increase in *ssb* expression is too low to be detected under the given experimental conditions. Similarly, moderately increased (~3.5 fold) *uvrA* expression was detected just in Courcelle et al. [30], whereas the other two studies reported no increase in *uvrA* expression [29,31]. Earlier, the induction of *uvrA* expression was reported to be about 4-fold, on average, as shown by the β-galactosidase assay [32].

On the other hand, we recently reported that *ssb* gene expression is indeed increased (more than two-fold) in γ-irradiated *E. coli*, as determined by a quantitative real-time PCR (RT-qPCR) analysis [33]. Hence, in this study we used the RT-qPCR and droplet digital PCR assays to determine the time course of *ssb* gene expression in various physiological contexts in *E. coli* to gain a deeper insight into the regulation of this essential gene.

## 2. Results

### 2.1. γ Irradiation Induces ssb and sulA Gene Expression

Recently, we reported the observation of the 2.5-fold increase in the *ssb* gene expression in γ-irradiated (400 Gy) wild-type *E. coli*, 40 min post irradiation at 37 °C [33].

In the current study we sought to characterize the expression of the *ssb* gene more thoroughly; hence, we determined its kinetics. Bacteria were irradiated with 400 Gy of γ-rays, incubated at 37 °C and samples taken 60 min post irradiation. In parallel, we determined the expression of the *sulA* gene, whose expression is one of the most heavily induced during SOS induction and is thus commonly used for quantifying the SOS response [18].

**Figure 2:**
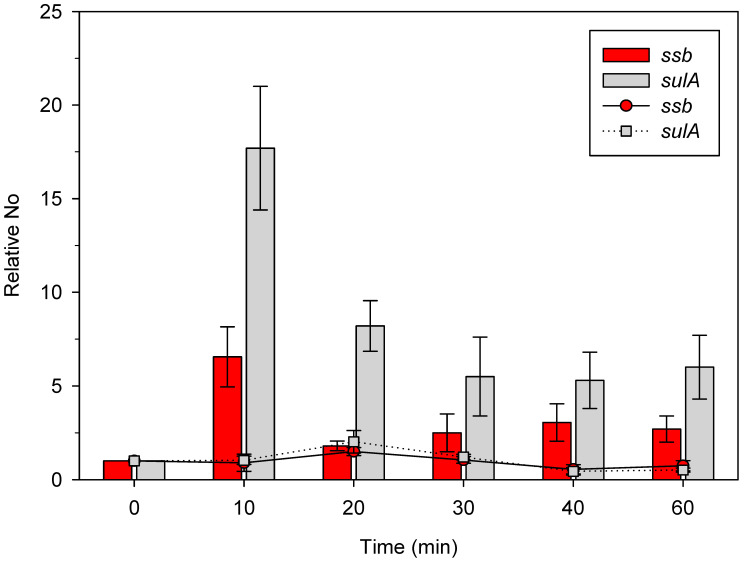
Expression of the *ssb* and the *sulA* gene in wild-type *E. coli* γ-irradiated with 400 Gy and incubated at 37 °C. Bacteria were irradiated when their exponential growth in LB medium reached OD_600_~0.4. Bars represent expression in irradiated bacteria (except at time 0), and dots and lines in unirradiated bacteria. No represents normalized average No value for the two genes. Each value is a mean of the three independent qRT-PCR experiments, with error bars representing standard deviation.

For reference, we used the expressions of the *ssb* and *sulA* genes in unirradiated bacteria, which were 9.4 × 10^−6^ ± 2.2 × 10^−6^ and 7.6 × 10^−6^ ± 2.7 × 10^−6^, respectively (*n* = 3). As shown in Figure 2, the expression of the *ssb* gene increased about 7-fold compared to an unirradiated control (shown as a bar at 0 min) 10 min after irradiation. After another 10 min of incubation, *ssb* expression decreased to the level about two-fold higher than the control (Figure 2). *ssb* gene expression was about three-fold higher in γ-irradiated bacteria 30 min, 40 min, and 60 min post irradiation, compared to the unirradiated control (Figure 2). This is in relatively good agreement with our previous results, wherein we observed about a 2.5-fold increase ine *ssb* expression 40 min post irradiation [33]. On the other hand, *sulA* gene expression increased more heavily than that of the *ssb* gene, being about 18-fold higher than the unirradiated control (shown as a bar at 0 min) and about 2.5-fold higher than the *ssb* gene 10 min post irradiation (Figure 2). Upon further incubation, *sulA* expression decreased about twice, to about 8 times the level of the unirradiated control 20 min post irradiation (Figure 2). The expression of *sulA* further decreased and stabilized at about five-fold above the control 30 min, 40 min, and 60 min post irradiation, which was about two-fold higher than the respective *ssb* gene expression (Figure 2). The presented results show that both *ssb* and *sulA* expression is increased in γ-irradiated bacteria, with the latter gene being induced more heavily.

As a control, we measured the expression of the two genes in the identical experimental setting, except for the application of no irradiation. As expected, no increase in *ssb* and *sulA* expression occurred in this situation (Figure 2), indicating that γ-irradiation induces both the *ssb* and SOS regulon (i.e., *sulA* gene).

Cell survival in γ-irradiated wild-type cultures was 0.22 ± 0.04 (*n* = 4), as compared to the unirradiated control.

### 2.2. UV Induces ssb and sulA Gene Expression

**Figure 3:**
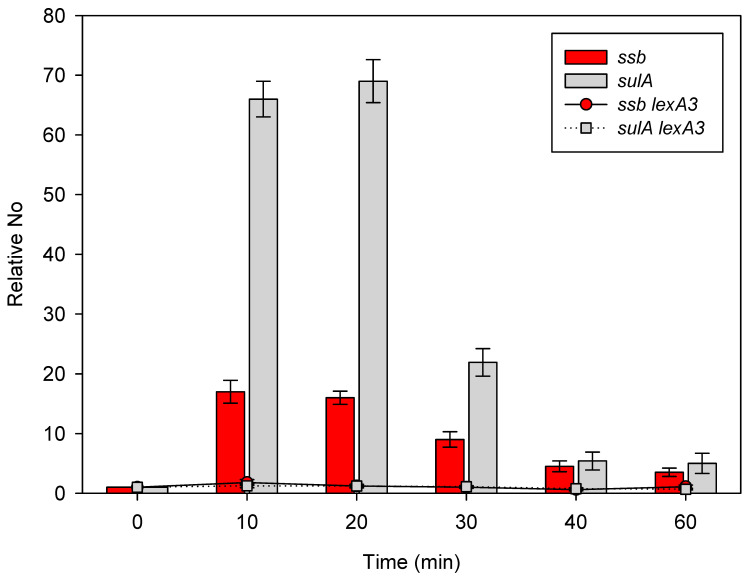
Expression of the *ssb* and the *sulA* gene in *E. coli* UV-irradiated with 40 Jm^−2^ and incubated at 37 °C. Bacteria were irradiated when their exponential growth in LB medium reached OD_600_~0.4. Data are shown for wild-type bacteria (bars) and *lexA3* (Ind^−^, SOS^−^) (dots and lines) mutants. Bars and dots at time 0 represent unirradiated control. No represents normalized average No value for the two genes. Each value is a mean of the three independent qRT-PCR experiments, with error bars representing standard deviation.

We irradiated bacteria with 40 Jm^−2^ of UV and followed the kinetics of *ssb* and *sulA* gene expression, as expressed in relation to the expressions of the two genes in unirradiated bacteria, which were 6.1 × 10^−6^ ± 1.8 × 10^−6^ and 7.2 × 10^−6^ ± 1.7 × 10^−6^, respectively (*n* = 3), and shown as bars at 0 min. The expression of *ssb* increased about 18- and 17-fold 10 min and 20 min after irradiation, respectively, as compared to the unirradiated control (Figure 2). Further incubation led to a decrease in the respective *ssb* expression to about 10-, 5-, and 4-fold above the control 30 min, 40 min, and 60 min post irradiation (Figure 3). Again, the expression of *sulA* increased more than *ssb*, reaching about 65- and 70-fold above the unirradiated control 10 min and 20 min post irradiation, respectively (Figure 3). Following further incubation, *sulA* expression dropped first to about 20-fold above the control 30 min post irradiation, and then to about 5-fold above the control 40 min and 60 min post irradiation (Figure 3).

Based on the above results, we can conclude that UV irradiation induces the expression of both *ssb* and *sulA* (i.e., SOS regulon).

Cell survival in UV-irradiated wild-type cultures was 0.86 ± 0.07 (*n* = 4) of that of the unirradiated part of the culture.

We also determined the expression profile of the *uvrA* gene in UV-irradiated bacteria, and showed that it was also induced in an SOS-dependent manner (Supplementary Figure S1).

### 2.3. Expression of the ssb Gene Is Regulated by the LexA Repressor

Our results have so far shown that both *ssb* and *sulA* (SOS regulon) are induced by UV- and γ-irradiation. In order to determine possible causality between the induction of the SOS and *ssb*, we measured the expression in an SOS uninducible *lexA3* mutant, which produces an uncleavable LexA repressor [18]. The *lexA3* mutant irradiated with γ-rays (not shown) or UV displayed no increase in the expressions of either the *ssb* or the *sulA* gene (Figure 3), compared to the unirradiated control, which were 2.1 × 10^−5^ ± 4.8 × 10^−6^ and 1.3 × 10^−5^ ± 2.9 × 10^−6^, respectively (*n* = 3).

The lack of *sulA* expression in SOS^−^ bacteria confirms its repression by the LexA repressor. However, much more interesting is the absence of *ssb* gene expression in SOS^−^ cells because it shows that the *E. coli ssb* gene is indeed under the control of the SOS regulon under stress conditions in bacteria.

### 2.4. The ssb Gene Is Overexpressed in Bacteria with Constitutively Activated SOS Regulon

We have shown that the *ssb* gene is induced when exogenous stress (UV or γ) activates the SOS response. Next, we explored whether SOS induction suffices for *ssb* gene induction, or the stressor is required. We measured *ssb* gene expression in an unirradiated *lexA71* (Def) mutant, which has inactive LexA repressor and thus the SOS system is constitutively induced [18].

Expression of the *ssb* gene was highly induced in the mutant during unimpeded exponential growth in a rich medium (OD_600_~0.4); its No value was 2.8 × 10^−2^ ± 1.1 × 10^−2^, while that of the wild-type control was 5.4 × 10^−5^ ± 3.2 × 10^−5^ (*n* = 3). Thus, the expression of the *ssb* gene was about 520-fold higher in the constitutively activated SOS mutant than in wild-type bacteria, without the application of any SOS inducing agent.

Hence, our results indicate that constitutive SOS activation suffices for the induction of *ssb* gene expression, whereas exogenous stress is dispensable.

### 2.5. ssb Gene Expression Is Unlinked to SOS Induction during Undisturbed Bacterial Growth

Considering the *ssb* promoters’ composition, which enables constitutive expression of this essential gene (see Introduction), we explored *ssb* gene expression during normal, unaffected growth of wild-type and *lexA3* mutant bacteria in rich medium with aeration, in mid-exponential, as well as in early and late stationary phases of growth. The expression of *sulA* was also determined, as a measure of SOS induction. To obtain more sensitive and reproducible data, we conducted an absolute quantification of transcripts by microfluidic droplet digital PCR (ddPCR).

**Figure 4:**
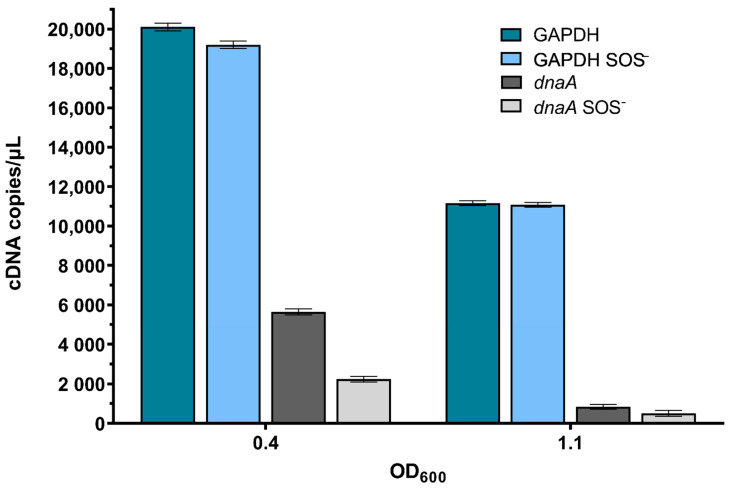
Absolute quantification of the GAPDH and *dnaA* genes’ transcripts during growth of wild-type and *lexA3* (SOS^−^) mutant bacteria in LB medium at 37 °C. Measurements were taken at mid-exponential growth phase (OD_600_~0.4) and early stationary growth phase (OD_600_~1.1). Three independent ddPCR experiments at three different concentrations gave the same relative results. Error bars represent Poisson confidence interval at a 95% level.

First, to check the sensitivity of the method as a measure of the overall level of gene expression in growing *E. coli* cultures, as well as its SOS dependence, we determined the expression of GAPDH (glyceraldehyde-3-phosphate dehydrogenase), a housekeeping gene unrelated to DNA metabolism, which is SOS independent; as well as the *dnaA* gene, which codes for the replication initiator protein, DnaA [34], and is SOS dependent [35]. GAPDH gene expression was very high in exponentially growing cells, at about 20,000 cDNA copies per µL, which fell about two-fold when cells reached the early stationary growth phase (Figure 4), and further diminished to about 140 cDNA copies per µL in late stationary phase cultures. GAPDH gene expression was at similar levels in wild-type and *lexA3* mutant bacteria, indicating that it is unaffected by the SOS regulon, which is unsurprising since this gene’s promoter does not possess an SOS box. Conversely, exponentially growing (OD_600_~0.4) wild-type and *lexA3* cells contained about 5600 and 2200 cDNA copies per µL of the *dnaA* transcript, respectively (Figure 4), indicating that the *dnaA* transcript level is increased in wild-type cells due to SOS induction. The *dnaA* transcript level fell more than 6-fold in the early stationary phase (OD_600_~1.1) in wild-type bacteria and about 5-fold in their *lexA3* derivatives (Figure 4), whereas in the late stationary phase cells, there was no *dnaA* expression.

**Figure 5:**
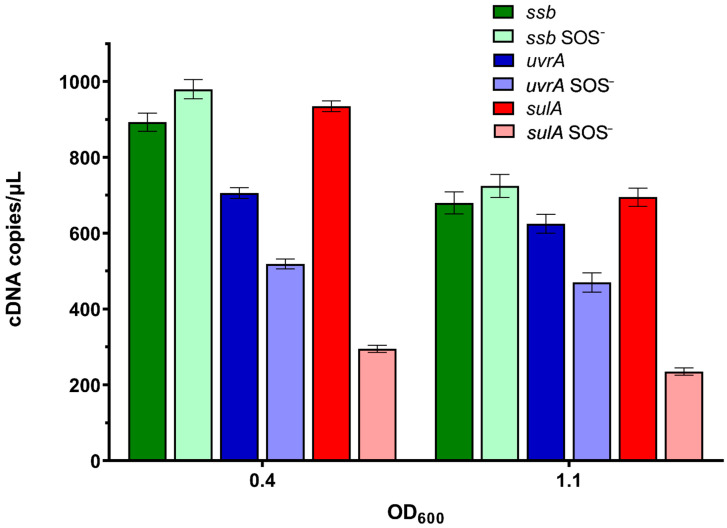
Absolute quantification of the *ssb*, *uvrA,* and *sulA* genes’ transcripts during growth of wild-type and *lexA3* (SOS^−^) mutant bacteria in LB medium at 37 °C. Measurements were taken at mid-exponential growth phase (OD_600_~0.4) and early stationary growth phase (OD_600_~1.1). Three independent ddPCR experiments at three different concentrations gave the same relative results (the presented results are with 24 ng cDNA/sample). Error bars represent Poisson confidence interval at a 95% level.

There were about 1000 cDNA copies per µL of the *ssb* gene transcript in both wild-type bacteria and their *lexA3* derivatives in the mid-exponential growth phase (OD_600_~0.4) (Figure 5). The level of the *ssb* transcript fell about 30% in both bacterial strains in the early stationary growth phase (OD_600_~1.1) (Figure 5). The data indicate a lack of SOS control of *ssb* gene expression.

In contrast, *uvrA* gene expression was about 700 cDNA copies per µL in the wild-type cells in the mid-exponential growth phase (OD_600_~0.4), whereas there were about thirty percent fewer transcripts in the *lexA3* mutant (Figure 5), indicating the SOS dependence of *uvrA* expression.

*sulA* gene expression reached about 900 cDNA copies per µL in wild-type cells in the mid-exponential growth phase (OD_600_~0.4), whereas there were about three-fold fewer transcripts in the *lexA3* mutant (Figure 5), suggesting that *sulA* is expressed at an elevated level due to SOS induction. In the early stationary growth phase (OD_600_~1.1), the expression of *sulA* decreased in both wild-type and *lexA3* bacteria, with the drop being larger in the former, thus reducing the difference in expression between the two bacterial strains (Figure 5).

## 3. Discussion

The SSB (RPA) class of proteins is essential for DNA metabolic processes in all living organisms. Although the *E. coli* SSB protein is the most thoroughly studied, and hence considered the prototypical bacterial SSB [1,2,3,4,13], regulation of its gene, *ssb*, is still poorly characterized (see Introduction). A complex structure of three *ssb* promotors, containing a single LexA repressor binding site (SOS box) [17], warrants a multifaceted expression profile, as we have reported here.

By using the RT-qPCR assay, we showed that the expression of *ssb* is induced by both UV- and γ-irradiation in a LexA-dependent manner, i.e., the gene is a part of the SOS regulon. Furthermore, we showed that SOS induction is sufficient for increasing the expression of *ssb*, while the application of an exogenous stressor is dispensable. Therefore, we were able to finally resolve the longstanding issue of the regulation of *ssb* expression by showing that it is indeed LexA dependent. The inability of earlier studies to detect an increase in *ssb* expression when the SOS response is activated can be attributed to the lower sensitivity of the assays used in those studies. The expression of *sulA* in UV-irradiated cells is indicative in this regard. In our assay (40 Jm^−2^ UV, rich medium), *sulA* expression rose ~70-fold compared to the unirradiated control, which lasted for ~20 min and then dropped to ~20-fold 30 min post irradiation and then to ~5-fold above the control level 40–60 min post irradiation (Figure 2). On the other hand, Courcelle et al. [30] (40 Jm^−2^ UV, minimal medium) observed a ~14-fold increase in *sulA* expression 5 min post irradiation, which fell to ~9-fold above the unirradiated control 60 min post irradiation. Quillardet et al. [31] (30 Jm^−2^ UV, rich medium) reported a ~7.5-fold increase in *sulA* expression compared to the unirradiated control 30 min post irradiation, which is about three-fold lower than our observation. Hence, although the experimental conditions were similar in the three studies, *sulA* expression was considerably higher in our report compared to the other two. Notably, the level of SSB is higher in cells grown in rich media than in those grown in minimal media [36]. In our assay, the increase in *ssb* expression was substantially lower than that of *sulA*, although the kinetics of both genes’ expressions are similar, with the highest increases observed early, then followed by gradual decreases while retaining the higher level compared to the unirradiated control even 60 min post irradiation. Consequently, lower *ssb* gene expression (compared to the *sulA* expression) combined with a lower sensitivity of microarray assays (compared to RT-qPCR) likely led to the lack of *ssb* gene induction detection in the previous two studies.

An interesting difference was observed in gene expressions in UV- and γ-irradiated bacteria. Namely, the peaks of the two genes’ expressions in UV-irradiated bacteria were about three-fold higher and lasted twice as long as those in γ-irradiated cells, although the γ survival was about four-fold lower than that of the cells irradiated with UV. These results suggest that UV is a more potent SOS inducer than γ-irradiation, which likely reflects the difference in the mechanism of SOS induction in the two situations, with DNA replication impairment being the main cause in the former and the processing of DNA double-strand breaks (DSBs) the main instigator of the SOS response in the latter [37,38,39]. A robust exonuclease activity of the RecBCD enzyme during DSB repair in wild-type cells may be regarded as a very strong suppressor of SOS induction as it thoroughly eliminates ssDNA tracts produced by its unwinding of the damaged DNA.

We revealed another level of *ssb* gene regulation while following its expression during bacterial growth in the absence of exogenous DNA damaging agents. First, we detected a gradient of gene expression in all tested genes depending on the bacterial growth phase. Namely, gene expression was highest in actively replicating and dividing bacteria during the exponential growth phase, which, in turn, decreased in parallel to the cessation of DNA replication and cell division. This is absolutely expected considering that each generation of dividing cells must duplicate their entire cellular structures.

However, we did notice some differences in the assayed genes’ expression profiles. *sulA* expression was higher in wild-type than in SOS^−^ cells, suggesting that the SOS regulon is induced in actively replicating and dividing bacteria. The difference in *sulA* expression between wild-type and SOS^−^ bacteria decreased in parallel with decreasing DNA replication and cell division (at OD_600_~1.1), indicating DNA replication as the likely cause of SOS induction. The SOS induction that we observed in undisturbed logarithmically growing wild-type *E. coli* is a remarkable result (that surely warrants further studying) that contradicts the commonly accepted knowledge that no SOS induction occurs during normal bacterial growth. Indeed, there is a report [40] of SOS induction (as determined by following the activity of the *sulA* promoter by GFP) in a small subpopulation (0.9%) of undisturbed log-phase wild-type cells. In comparison, our observation of a ~3-fold increased *sulA* expression level in wild-type vs SOS^−^ culture is certainly more physiologically relevant. For, if ~1% of the cells produce that ~3-fold increase in the whole bacterial population (as we measured), their *sulA* expression should have increased ~300-fold, which is highly unlikely considering that those cultures were undisturbed and such a strong *sulA* induction was not observed even in heavily disturbed bacteria. Consequently, our data suggest at least an order of magnitude higher incidence of SOS-induced cells in an assayed population, compared to the previous report. Notably, bacteria were grown in minimal medium in the previous study, whereas we grew them in rich medium. Therefore, the application of the highly sensitive ddPCR assay revealed a surprisingly high induction of the SOS regulon in the undisturbed wild-type log-phase-grown *E. coli* population, which may have a strong ramification for our perception of the physiological role of the SOS system in bacteria.

A similar, though a bit less pronounced, SOS dependence was observed in the expression of the *dnaA*, a gene coding for the replication initiator protein DnaA. Although the expression of *E. coli dnaA* is known to be induced by exogenous stress in an SOS-dependent way [35], this is the first report of SOS control of this gene expression during normal, undisturbed bacterial log-phase growth, which is, incidentally, enabled by DnaA. This in turn raises the question of the possible SOS control of DNA replication (initiation) in wild-type bacteria grown in undisturbed conditions. Notably, *dnaA* overexpression in exponentially growing wild-type bacteria may limit SOS induction, as proposed [41].

On the other hand, *ssb* expression was more complex, which is not surprising considering that this essential gene harbors multiple promoters. Namely, no difference was noted in *ssb* gene expression in wild-type and SOS^−^ cells in undisturbed, logarithmically-grown cultures, although the increased expression of *sulA* in the former shows that the SOS regulon was indeed induced. One is tempted to conclude that *ssb* gene expression is independent of the SOS regulon during the normal growth of *E. coli*, but *ssb* overexpression in undisturbed bacteria with a constitutively induced SOS regulon argues against such an explanation. In addition, the (moderate) SOS-dependence of *uvrA* gene expression, which is repressed by the same SOS box as *ssb*, reveals fine differences in the regulation of the two genes. The LexA box that controls both genes is quite heterologous (HI 6.98) to the consensus SOS-box sequence, and is thus bound by the LexA repressor with lower affinity [19]. The consequence of this is the relatively high basal expression of both genes, which does not increase substantially (or at all) upon moderate SOS induction. The different expressions of *ssb* and *uvrA* in logarithmically-grown cells can be explained by considering their position in respect to the LexA box. Namely, the SOS box is quite distant (170 nt) from the *ssb* coding region and its control is restricted to the most distant (of the three) *ssb* promoter [17], whereas the LexA box overlaps with the −35 region of the *uvrA* promotor. Consequently, LexA repressor inactivation affects *uvrA* expression more directly than that of *ssb*.

Our results thus indicate that *ssb* gene expression is quite stringently suppressed by the LexA repressor in wild-type cells; so much so that the basal *ssb* gene expression is elevated only with stronger SOS induction, such as after UV- and γ-irradiation, or due to constitutive SOS induction. Weaker SOS induction, such as that observed during normal growth in a rich medium, is obviously insufficient for increasing *ssb* expression. On the other hand, the basal *ssb* expression level is quite high in undisturbed growing *E. coli*: it is as high as that of induced *sulA*. Therefore, we conclude that the expression of the essential *E. coli ssb* gene is SOS regulated in such a way that its basal level is relatively high, whereas stronger SOS induction is required for an increase in *ssb* expression. The basal level of SSB proteins in exponentially growing wt *E. coli* in LB medium reaches ~8000 copies, e.g., ~2000 tetramers [42], while its consumption during replication should not exceed 200 tetramers [43]. The excess ~1800 tetramers are mostly membrane-localized, from which they swiftly translocate to DNA following DNA damage [43]. Therefore, a combination of the previous results concerning SSB concentration in a cell, and our present results, indicates that even basal *ssb* gene expression produces a considerable SSB surplus (~1800 tetramers) in an undisturbed bacterial population grown exponentially in a rich medium, and thus there is no need for induced *ssb* gene expression in those bacteria.

## 4. Materials and Methods

### 4.1. Strains, Growth Conditions, and Media

All strains used were derivatives of wild-type *E. coli* K-12 strain AB1157 [44]. The following mutants were constructed by P1 transduction, as described earlier [45]: DE127 *lexA3* (Ind^−^, uncleavable LexA repressor) *malB*::Tn*9* [33]; DE675 *lexA71*::Tn*5* (Def, inoperative LexA repressor) Δ*sulA*::FRT (laboratory collection). Bacteria were grown at 37 °C in rich Luria–Bertani (LB) broth, with aeration, and on LB plates [45].

### 4.2. Cell Irradiation by UV and γ-Rays

A fresh overnight bacterial culture was diluted 100-fold in fresh LB broth and grown with aeration at 37 °C until reaching an optical density of 0.4 at 600 nm (OD_600_).

For UV irradiation, a bacterial culture was spread into Petri dishes so that they covered the bottom as a no more than 2-mm-thick layer. The bacteria were irradiated with a low-pressure Hg germicidal lamp, which produced UV of 254 nm at a rate of 2 Jm^−2^ s^−1^. Irradiation was performed at room temperature while mixing bacteria, and then the irradiated cells were transferred into an Erlenmeyer flask and incubated at 37 °C in the dark. Samples were collected at 10, 20, 30, 40, and 60 min after irradiation.

Bacteria were γ-irradiated using a ^60^Co source, with a dose of 400 Gy, at a dose rate of 2 Gy s^−1^. A bacterial culture was γ-irradiated at 0 °C in an Erlenmeyer flask and then incubated at 37 °C and samples taken at 10, 20, 30, 40, and 60 min after irradiation.

For survival assays, irradiated bacterial cultures were serially diluted in 67 mM phosphate buffer (pH 7.0), and aliquots spread on LB plates, which were then incubated at 37 °C for 24–48 h. Cell survival was expressed as a fraction of unirradiated control, and expressed as an average value from three experiments ± standard deviation.

### 4.3. RNA Isolation and Reverse Transcription

Bacterial RNA was extracted from 8 mL samples of bacterial cultures using the RNeasy^®^ Plus Mini Kit (Qiagen, Hilden, Germany) and RNAprotect™ Bacteria Reagent (Qiagen) according to the manufacturer’s instructions. Integrity of RNA was checked by gel electrophoresis, while its quantification was performed with the Quant-IT RNA assay Kit using Qubit fluorometer (Invitrogen, Waltham, MA, USA). Approximately 0.5 μg of RNA was reverse transcribed by the PrimeScript RT reagent kit with genomic DNA Eraser (perfect Real Time, Takara, Mississauga, ON, Canada) using random and oligo dT primers mix in 10 μL reaction. Negative controls without reverse transcriptase were used for all samples.

### 4.4. Quantitative Real-Time PCR (RT-qPCR) Analysis

Primers for the *ssb* and *sulA* gene expression analysis by RT-qPCR were: fw-GTTGTGCTGTTCGGCAAACT and rev-GCGATCCTGACCGGATTGAT; fw-GCCGGGCTTATCAGTGAAGT, and rev-CCTGAACCCATTCCCGACTC, respectively. Endogenous control genes for normalization were: 16S ribosomal gene, fw-GTTAGCCGGTGCTTCTTCTG and rev-CAGCCACACTGGAACTGAGA; and glyceraldehyde-3-phosphate dehydrogenase (GAPDH) gene, fw-GGAACGCCATACCAGTCAGT and rev-AGGTCTGATGACCACCGTTC. Both endogenous controls gave the same results, and we used the latter as it is commonly used as a control and, as shown in Figure 4, its expression is SOS independent. Additionally, Figure 4 shows that GAPDH expression varies less than 2-fold in different bacterial growth phases, which is all indicative of its suitability as a control. We used the following thermal cycling conditions: 50 °C 2 min, 95 °C 7 min, 95 °C 15 s, 60 °C 1 min for 40 cycles followed by dissociation stage: 95 °C for 15 s, 60 °C for 1 min, 95 °C for 15 s, and 60 °C for 15 s. We tested specificity of amplified products on agarose gel and confirmed them by dissociation curve analysis. For post-run data analysis, we used LinRegPCR software v.11.1 [46,47], which enables calculation of the starting concentration of amplicon (“No value”). “No value” is expressed in arbitrary fluorescence units and is calculated by considering PCR efficiency, ranging between 90–100%, and baseline fluorescence. “No value” determined for each technical replicate was averaged and such averaged “No values” were divided by “No values” of endogenous control. Normalized “No values” were compared using unpaired t-test, which compares the mean of two unmatched groups.

### 4.5. Absolute Quantification of RNA Levels by Microfluidic Digital PCR

Microfluidic droplet digital PCR (ddPCR) procedure was performed using the QIAcuity 2-plex instrument (Qiagen, Hilden, Germany). The ddPCR reaction mixture was assembled using QIAcuity 3X Eva Green PCR Master Mix, 10X primer mix (4 μM), RNase-free water, and a fixed concentration of cDNA template in a final volume of 15 μL per sample. After accurate vortexing, 12 μL of the above prepared mixture was transferred into the 24-well 8.5 K nanoplate and sealed with the nanoplate seal. For quality control of QIAcuity dPCR, the assay was replicated with different amounts of template input (24, 12, and 6 ng). The sequence of primers for transcript detection of *ssb*, *sulA*, and GAPDH genes was the same as that used in RT-qPCR; primers used for *dnaA* gene were: fw-ATCATTCTCACCTCGGATCG and rev-AGACGCTGGCGATAAAGAA. Considering the *uvrA* gene is 2823 nt long, we used two sets of primers: fw-GCAGGAGAAAGCCCCTCAAT/ rev-TCGGGTTCTGGCAAATCCTC and fw-ACGACGAATGGAGGTATCGC/ rev-CAGATGCTGAAATCGCTGGC. The results were very similar and only those obtained with a first set of primers are shown. The 8.5 K nanoplate gives rise to 8500 single partitions in which the template is distributed randomly. The QIAcuity carries out fully automated processing including all necessary steps for plate priming, sealing of partitions, thermocycling, and image analysis. The amplification cycling protocol include 95 °C for 2 min for enzyme activation and the following 40 cycles of 15 s at 95 °C for denaturation, 15 s at 60 °C for annealing, and 15 s at 72 °C for extension, and then a final step at 40 °C for 5 min. Fluorescence light is emitted by positive partitions that have a target molecule, as compared to those without target, the negative partitions. The experiments were performed using a negative control without reverse transcription enzyme. Data were analyzed using the QIAcuity Suite Software V1.1.3 (Qiagen) and the results are expressed in copies of cDNA/µL based on Poisson statistics analyses. The partitions produced by the machine resemble Poisson process since the targets finish in partitions independently and with a fixed rate. The Poisson distribution gives probabilities for positive integer random events. The parameter of this distribution, λ, is the expectation value for these events, which means it is the mean probability for a proportion of a counting process or the counting process for the ddPCR analysis. Furthermore, the QIAcuity has imbedded software that can quantify and produce reliable statistics. In our case the statistical measure we considered was the Poisson confidence interval at a 95% level that, when plotted, shows whether the events differ with 95% confidence.

## Data Availability

Not applicable.

## Supplementary Materials

The following supporting information can be downloaded at: https://www.mdpi.com/article/10.3390/ijms231810917/s1.
