# Supplementary material for: Regulation of ssb Gene Expression in Escherichia coli
Source: Int J Mol Sci. 2022 Sep 18;23(18):10917. doi: 10.3390/ijms231810917 (PMC9505508; doi:10.3390/ijms231810917)
Supplement: Supplementary file 1 [file ijms-23-10917-s001.zip › ijms-1880677-supplementary.pdf]

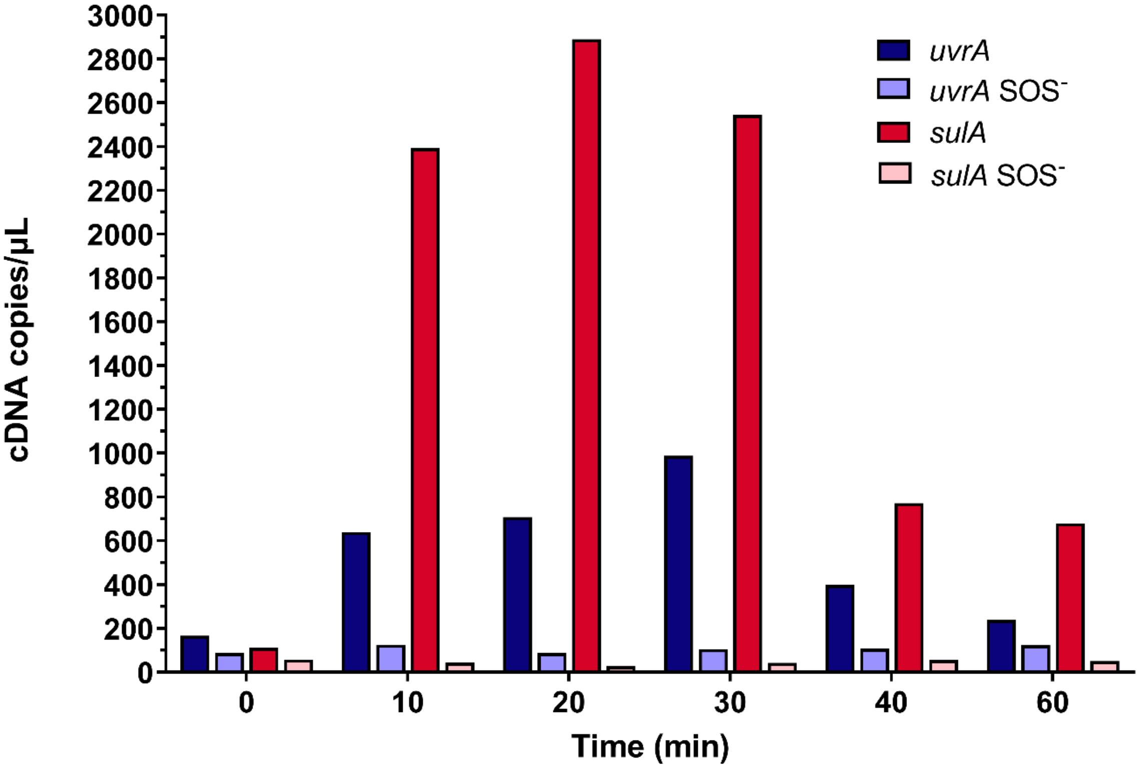

Supplementary Figure S1. Absolute quantification of expression of the *uvrA* and the *sulA* gene in *E. coli* UV-irradiated with 40 Jm^{-2} and incubated at 37 ^{o}C. Bars at time 0 represent unirradiated control. Three independent ddPCR experiments at three different concentrations gave the same relative results (the presented results are with 6 ng cDNA/sample). Poisson confidence interval at a 95% level is < 1%.
